# MTPC integration for improved diagnosis of pleural effusion

**DOI:** 10.3389/fonc.2025.1629939

**Published:** 2026-01-16

**Authors:** Shuai Zhao, Jiajia Liu, Jing Chen, Ji Shi, Ming Ma, Haiyang Yan, Jingna Sun, Yan Geng

**Affiliations:** 1 Xi’an Jiaotong University, Xi’an, Shaanxi, China; 2 Department of Clinical Laboratory, The First Hospital of Hebei Medical University, Shijiazhuang, Hebei, China; 3 Department of Anesthesiology, The First Hospital of Hebei Medical University, Shijiazhuang, Hebei, China; 4 Department of Clinical Laboratory, The Fourth Hospital of Hebei Medical University, Shijiazhuang, Hebei, China; 5 Clinical Laboratory, The Second Affiliated Hospital of Xi’an Jiaotong University, Xi’an, Shaanxi, China

**Keywords:** DNA ploidy analysis, gene methylation, MTPC panel, pleural effusion, tumor markers

## Abstract

**Introduction:**

Pleural effusion is clinically common with diverse etiologies, and differentiating benign from malignant cases is critical for treatment planning and prognosis assessment. Traditional single diagnostic methods have inherent limitations, leading to diagnostic challenges. This study aimed to develop a multi-modal diagnostic panel (MTPC) to improve the accuracy and efficiency of initial pleural effusion diagnosis.

**Methods:**

A total of 369 patients (264 with malignant pleural effusion and 105 with benign pleural effusion) were enrolled retrospectively. The MTPC panel integrated four diagnostic modalities: methylation biomarkers (PTGER4 and SHOX2), tumor markers (CEA and CYFRA21-1), DNA ploidy analysis, and cytological examination. Diagnostic performance was evaluated using sensitivity, specificity, and area under the receiver operating characteristic curve (AUC). Additional analyses were performed for cytology-undetermined and cytology-negative cases.

**Results:**

Among immunological tumor markers, CEA exhibited the highest specificity (98.1%) and CYFRA21-1 the highest sensitivity (56.8%). Combined PTGER4 and SHOX2 methylation detection achieved a sensitivity of 65.9% and specificity of 92.4%. The MTPC panel demonstrated the best diagnostic performance, with an AUC of 0.8698, sensitivity of 90.2%, and specificity of 83.8%. In cytology-undetermined cases, MTPC reduced “cytology undetermined” reports by 78.4% and missed diagnoses via “negative” reports by 92.3%.

**Discussion:**

The MTPC panel effectively integrates molecular, immunological, chromosomal, and cytomorphological data, significantly improving the diagnostic efficiency of pleural effusion. It addresses the limitations of single diagnostic methods and provides more reliable evidence for clinicians, facilitating early and accurate differentiation of benign and malignant pleural effusions.

## Introduction

Pleural effusion is a common clinical condition with diverse and complex etiologies. It can be caused by various factors such as infections, malignancies, and cardiac or liver diseases. Patients often present with symptoms like chest pain, shortness of breath, and cough. Accurately differentiating between benign and malignant pleural effusions is crucial for formulating subsequent treatment strategies and evaluating the prognosis of patients (1, 2). However, when patients first present with pleural effusion, it is often difficult to provide timely and targeted treatment due to unclear pathological diagnoses. This is because pathological diagnosis of pleural effusion faces numerous challenges. Even in pleural effusions caused by malignancies, factors such as tumor heterogeneity, a low number of cells in the effusion, and atypical cell morphology make it challenging to obtain a definite diagnosis from the first specimen submitted for examination. This diagnostic dilemma is more prominent in primary care hospitals, where pathologists may have relatively less experience.

Epigenetic methylation detection has emerged as a research hotspot in the field of tumor diagnosis in recent years. In China, over the past decade, 31 tumor gene methylation detection products have obtained NMPA (National Medical Products Administration) approval, covering various common malignancies such as colorectal cancer, lung cancer, liver cancer, and cervical cancer. Most of these products screen or triage patients at high risk of tumors by detecting specific methylated markers in cell-free DNA in peripheral blood. If it is possible to obtain cell or tissue samples from the diseased site and then detect the methylation of tumor suppressor genes, it may improve the specificity and accuracy of diagnosis (3). The study by Zhang et al. demonstrated that after precise sampling of lung lesions using bronchoscopy, the sensitivity of methylation detection could reach up to 81.9%, with a specificity of 87.1% (4). The exfoliated cells in pleural effusion mostly originate from diseased sites of tissues and organs such as the lungs, pleura, and esophagus. The genomic DNA in these cells can better reflect the epigenetic changes of patients and is suitable for gene methylation detection.

Immunological tumor markers play a vital role in the diagnosis, treatment, and prognosis assessment of tumors. Immunological tumor markers are substances produced and released by tumor cells or generated when the body mounts an immune response to tumors. They can be detected in blood, body fluids, and tissues. Representative immunological tumor markers such as carcinoembryonic antigen (CEA), neuron-specific enolase (NSE), progastrin-releasing peptide (proGRP), cytokeratin 19 fragment (CYFRA21-1), and squamous cell carcinoma antigen (SCC) have been widely used in clinical practice. However, they have not been used for the auxiliary diagnosis of pleural effusion in clinical practice. The study by Fan et al. showed that immunological tumor markers can be detected in pleural effusion, but they have not been applied in large-scale clinical practice (5).

DNA ploidy analysis provides a diagnostic basis at the chromosomal level. Normal cells have a stable DNA content and are in an euploid state. However, during the proliferation of tumor cells, due to abnormal gene regulation and errors in chromosome segregation, changes in DNA content often occur, presenting as aneuploidy. By analyzing the DNA ploidy of cells in pleural effusion, it is possible to determine the proliferation activity of cells and whether malignant transformation has occurred (6, 7). Nevertheless, the sensitivity of DNA ploidy analysis is relatively low, and its use alone has certain limitations.

**Figure 1:**
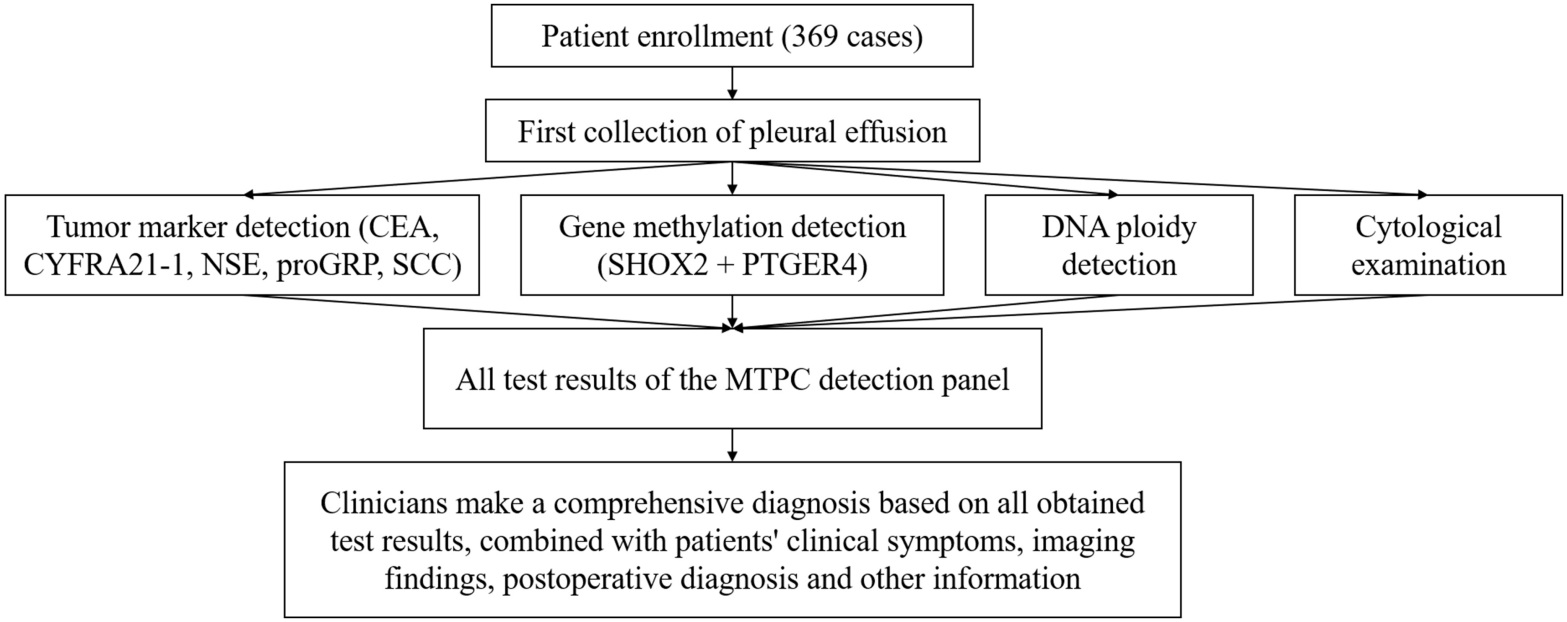
Flowchart of the MTPC panel workflow for pleural effusion diagnosis.

Given the limitations of traditional single diagnostic methods, a MTPC panel strategy has emerged. This study innovatively proposes a diagnosis panel named MTPC for pleural effusion. It simultaneously performs PTGER4 and SHOX2 gene methylation detection (M, methylation), CEA and CYFRA21–1 tumor marker detection (T, tumor markers), DNA ploidy analysis (P, ploidy), and cytological examination (C, cytology) on pleural effusion. Compared with conventional cytological diagnosis of pleural effusion, the MTPC panel innovatively integrates multi-dimensional clinical information at the molecular, immunological, chromosomal, and cytomorphological levels to conduct comprehensive and multi-level analysis of pleural effusion. This effectively improves the sensitivity and accuracy of pleural effusion diagnosis and provides more reliable diagnostic evidence for clinicians (**Figure 1**). It addresses the clinical dilemma where clinicians previously could only determine the nature of pleural effusion based on cytomorphological results, and offers a novel diagnostic approach that helps patients clarify the benign or malignant nature of pleural effusion at an early stage from a more microscopic and objective perspective.

## Methods

### Patients and pleural effusion collection

In this study, we retrospectively collected the test results of pleural effusion samples from patients hospitalized in the Department of Respiratory Medicine and Department of Thoracic Surgery of our hospital between October 2022 and November 2024. A total of 369 patients were enrolled, including 264 with confirmed malignant pleural effusion and 105 with benign pleural effusion. When collecting pleural effusion, the patient was placed in a sitting or semi-recumbent position. After disinfection, draping, and anesthesia, a needle was inserted along the upper edge of the rib for puncture. The first aspiration volume did not exceed 600 ml, and the sample was collected. After the procedure, some samples were sent to the Pathology Department for cytomorphological pathological diagnosis, and some were sent to the Clinical Laboratory Department for tumor marker, gene methylation detection, and DNA ploidy analysis.

To minimize incorporation bias, this study only enrolled patients with pleural effusion who had not received a confirmed diagnosis before hospitalization, and consecutive sampling was conducted. All samples were the first-collected pleural effusion from patients. When the Pathology Department performed cytological diagnosis and the Clinical Laboratory Department conducted the other three tests, the test results were not shared in advance between the two departments. All tests conducted by the Clinical Laboratory Department generated results on automated equipment, and the cut-off values used were those recommended by the manufacturers following their NMPA registration. The diagnostic results were based on the final clinical diagnosis documented in the patients’ discharge certificates.

A positive result in any one of the four diagnostic modalities—gene methylation detection, tumor marker detection, DNA ploidy analysis, and cytological diagnosis—defines a positive outcome for the MTPC panel (**Figure 1**). This is because each diagnostic method is relatively independent, covering four distinct dimensions: genome, protein, chromosome, and cell. The combined application can compensate for disparities in diagnostic efficacy among different methods. In this study, analytical methods such as logistic regression were not employed, primarily due to the qualitative nature of all test results; the information density of the independent variables was insufficient for logistic regression analysis. Therefore, the data analysis mainly relied on univariate analysis and receiver operating characteristic (ROC) curves.

In this study, gene methylation, immunological tumor marker, and DNA ploidy detection of patients’ pleural effusion were only performed on the first collected sample. The final clinical diagnosis of patients was based on the discharge medical records. Specifically, if tumor cells were detected in the pleural effusion cytological diagnosis, the final diagnosis was confirmed as malignant pleural effusion. In contrast, if no tumor cells were found in the cytological diagnosis, the patient’s final diagnosis was determined by clinicians through a comprehensive assessment of all patient information, including pathological results from possible needle biopsies or surgical resections.

### DNA extraction, bisulfite treatment, and methylation detection

The procedures for DNA extraction and bisulfite purification were consistent with previous reports. The kits were purchased from Beijing Jiaheng Yongtai Technology Co., Ltd (8). After purification, the bisulfite-converted DNA was amplified in parallel tubes by multiple methylation-specific real-time PCR (MS-PCR). MS-PCR amplified methylated SHOX2 (VIC), PTGER4 (FAM), and β-ACTB (CY5) DNA, with β-ACTB serving as an internal control for quantifying the total input DNA. PCR amplification targeting sequences modified with sodium bisulfite was detected using TaqMan probes. The reaction volume was 40 μL, containing 5 μL of bisulfite-modified DNA, 250 μM dNTPs, 0.8 μL of each primer (10 μM), 1.5–3 mM MgCl_2_, 20 μL of 2×Taq buffer (including dNTPs and Taq polymerase), and 13.4 μL of double-distilled water (ddH_2_O). The reaction was carried out in a thermocycler with the following cycling parameters: 95°C for 10 min; 45 cycles of 95°C for 30 s; a specific annealing temperature of 58°C for 35 s, 72°C for 30 s; and a final extension step at 72°C for 8 min.

Sulfite was used to modify unmethylated cytosine bases to uracil bases, which were then converted to thymine bases during PCR amplification. Methylated cytosine bases remained unchanged, enabling the differentiation between methylated and unmethylated cytosine bases. Specific primers targeting sequences after bisulfite modification were designed for PCR. PCR amplification products were detected using Taq-Man probes (85 nM). A plasmid containing the corresponding methylated gene sequence was used as the positive control, and purified water was used as the negative control. The β-actin gene served as the internal control for quantifying the total input DNA. The baseline position was adjusted (Threshold = 10000; Noise band = 0.8) to obtain the Ct value of the fluorescence signal. The Ct value of the β-actin gene signal should be between 18 and 32. When performing multi-gene combined detection, since the functions of each gene were independent of each other, if any one gene was positive, the result of the combined test was judged as positive. As a product certified by NMPA in China, the cut-off value was established based on the verification of a large number of clinical samples by regulatory authorities.

### Tumor marker detection of pleural effusion

At the clinical laboratory department, the levels of CEA, NSE, Pro-GRP, CYFRA 21-1, and SCC were detected using commercial mature test assays (Tellgen, Shanghai, China) according to the manufacturer’s instructions, with a TESMI analyzer. The cut-off value was determined based on the group standard jointly developed by our institution and the Tuberculosis Diagnosis and Treatment Technology Innovation Alliance in 2022.

### DNA ploidy analysis of pleural effusion

All slides stained with Feulgen were analyzed using the MotiCytometer automatic cell image analysis system manufactured by Motic Medical Diagnostic Systems Co., Ltd., Xiamen. The pleural fluid cells were centrifuged to form a pellet. After treatment with the cell preservation solution, Feulgen staining was performed for DNA ploidy analysis. When more than three cells had a DNA index ≥ 2.5 or aneuploid cells were observed, the specimen was diagnosed as positive.

### Pathological diagnosis of pleural effusion

For each patient, pleural effusion samples were obtained by pleural puncture and chest drainage. After centrifugation at 2000g for 10 minutes, the supernatant was carefully transferred to a new tube without disturbing the sediment. The sediment was used for liquid-based cytology testing or made into paraffin-embedded cell blocks and stained with hematoxylin-eosin. At the same time, immunocytochemical staining examination was performed on the cell-block sections as a supplement. All slides were reviewed separately by two senior pathologists. Detecting pathological changes of malignant tumors in pleural biopsy tissues or cytology has always been regarded as the gold standard for diagnosing malignant pleural effusion.

The results of cytological diagnosis are classified into three categories: positive, indicating the definite detection of malignant tumor cells; negative, indicating the definite absence of malignant tumor cells; and cytology undetermined, indicating the detection of suspicious or atypical cells. A positive result from cytological diagnosis serves as a basis for determining a positive outcome of the MTPC panel; meanwhile, a positive cytological result is also the most clinically directive basis for diagnosis. When calculating sensitivity and specificity, both cytology negative and cytology undetermined were regarded as negative results. This is because clinicians cannot formulate effective treatment plans for patients based on cytology undetermined results.

### Statistical analysis

Statistical analysis was performed using the SPSS 19.0 software package (SPSS Inc., Chicago, IL). A chi-squared test was used to analyze the methylation frequency of SHOX2 and PTGER4 genes, as well as tumor markers, DNA ploidy analysis, and cytological examinations. The receiver operating characteristic curve (ROC) was used to calculate the area under the ROC curve (AUC) to evaluate the diagnostic effect. Statistical significance was set at a P value < 0.05.

### Ethical statement

All methods were carried out in strict accordance with relevant guidelines and regulations. All experimental protocols were approved by the Clinical Research Ethics Committee of the First Hospital of Hebei Medical University (Ethics Approval No. S00382).

## Results

### Characteristics of patients with malignant and benign pleural effusions

This study enrolled 264 patients with malignant pleural effusion and 105 benign pleural effusion, as shown in **Table 1**. Among the patients with malignant pleural effusion, 187 had lung adenocarcinoma (LUAC), 21 had lung squamous cell carcinoma (LUSC), 10 had small cell carcinoma (SCLC), 14 had pleural mesothelioma (PM), 1 had sarcomatoid carcinoma (SC), and 31 had unclassified lung cancer. In the benign pleural effusion group, 67 had pulmonary tuberculosis (PTB) and 38 had non-tuberculous infections. The average age of patients with malignant pleural effusion was 59 years old, with 51.9% being over 60 years old, and the male proportion was 50.4%. The average age of the benign pleural effusion group was 56 years old, with 47.6% being over 60 years old, and the male proportion was 73.3%, which was significantly higher than that of female patients (χ²=22.86, p < 0.001).

**Table 1:** Distribution of basic characteristics of patients with malignant pleural effusion and the benign pleural effusion group.

Category		Malignant pleural effusion		Benign pleural effusion	
		n	%	n	%
Total		264		105	
Age					
	<40	18	6.8%	21	20.0%
	40-59	109	41.3%	34	32.4%
	≥60	137	51.9%	50	47.6%
	Age range	24-83		17-86	
	Median age	59		56	
Sex					
	Male	133	50.4%	77	73.3%
	Female	131	49.6%	28	26.7%
Histology subtype					
Malignant pleural effusion					
	LUAC	187	70.8%		
	LUSC	21	8.0%		
	SCLC	10	3.8%		
	PM	14	5.3%		
	SC	1	0.4%		
	Unclassified Lung Cancer	31	11.7%		
Benign pleural effusion					
	PTB			67	63.8%
	Non-tuberculous infection			38	36.2%

### Detection results of immunological tumor markers in pleural effusion samples

In this study, five tumor markers, including carcinoembryonic antigen (CEA), neuron-specific enolase (NSE), progastrin-releasing peptide (proGRP), cytokeratin 19 fragment (CYFRA21-1), and squamous cell carcinoma antigen (SCC), were detected in pleural effusion samples. Among them, CEA had the highest diagnostic specificity of 98.1%, while NSE had the lowest specificity of 74.3%. The specificities of proGRP, CYFRA21-1, and SCC were 89.5%, 88.6%, and 85.7%, respectively. When immunological tumor markers were used for the diagnosis of pleural effusion, the sensitivities from high to low were CYFRA21-1, CEA, proGRP, NSE, and SCC (56.8%, 30.3%, 22.0%, 20.8%, and 11.7%, respectively). For malignant pleural effusions caused by different pathological types of tumors, CEA and CYFRA21–1 had the highest sensitivity for pleural effusions caused by lung adenocarcinoma (35.8% and 60.4%, respectively), NSE and proGRP had the highest sensitivities for pleural effusions in patients with small cell carcinoma (50.0% and 60.0%, respectively), and SCC had the highest sensitivity for pleural effusions in patients with lung squamous cell carcinoma, which was 47.6% (**Table 2**).

**Table 2:** Sensitivity and specificity of immunological tumor markers in the auxiliary diagnosis of pleural effusion in different types of malignant pleural effusions and the benign pleural effusion group.

Histology subtype			CEA			NSE			proGRP			CYFRA21-1			SCC		
		Total	n	Sensitivity	Specificity	n	Sensitivity	Specificity	n	Sensitivity	Specificity	n	Sensitivity	Specificity	n	Sensitivity	Specificity
Malignant pleural effusion																	
LUAC		187	67	35.8%		35	18.7%		36	19.3%		113	60.4%		16	8.6%	
LUSC		21	5	23.8%		4	19.0%		4	19.0%		11	52.4%		10	47.6%	
SCLC		10	2	20.0%		5	50.0%		6	60.0%		2	20.0%		0	0.0%	
PM		14	0	0.0%		3	21.4%		4	28.6%		10	71.4%		2	14.3%	
SC		1	0	0.0%		0	0.0%		0	0.0%		0	0.0%		0	0.0%	
Unclassified Lung Cancer		31	6	19.4%		8	25.8%		8	25.8%		14	45.2%		3	9.7%	
Total		264	80	30.3%		55	20.8%		58	22.0%		150	56.8%		31	11.7%	
Benign pleural effusion																	
PTB		67	1	1.5%	98.5%	13	19.4%	80.6%	9	13.4%	86.6%	5	7.5%	92.5%	11	16.4%	83.6%
Non-tuberculous infection		38	1	2.6%	97.4%	14	36.8%	63.2%	2	5.3%	94.7%	7	18.4%	81.6%	4	10.5%	89.5%
Total		105	2	1.9%	98.1%	27	25.7%	74.3%	11	10.5%	89.5%	12	11.4%	88.6%	15	14.3%	85.7%

Sensitivity = (Number of patients with positive detection of a specific tumor marker in a particular type of malignant pleural effusion/Total number of patients with that type of malignant pleural effusion) × 100%.

Specificity = (Number of patients with negative detection of a specific tumor marker in benign pleural effusion/Total number of patients with benign pleural effusion) × 100%.

LUAC, lung adenocarcinoma; LUSC, lung squamous cell carcinoma; SCLC, small cell lung cancer; PM, pleural mesothelioma; SC, sarcomatoid carcinoma; PTB, pulmonary tuberculosis.

The sample sizes of the LUSC, SCLC, PM, and SC subgroups are relatively small, so their analytical results may be biased and should be interpreted with caution.

**Figure 2:**
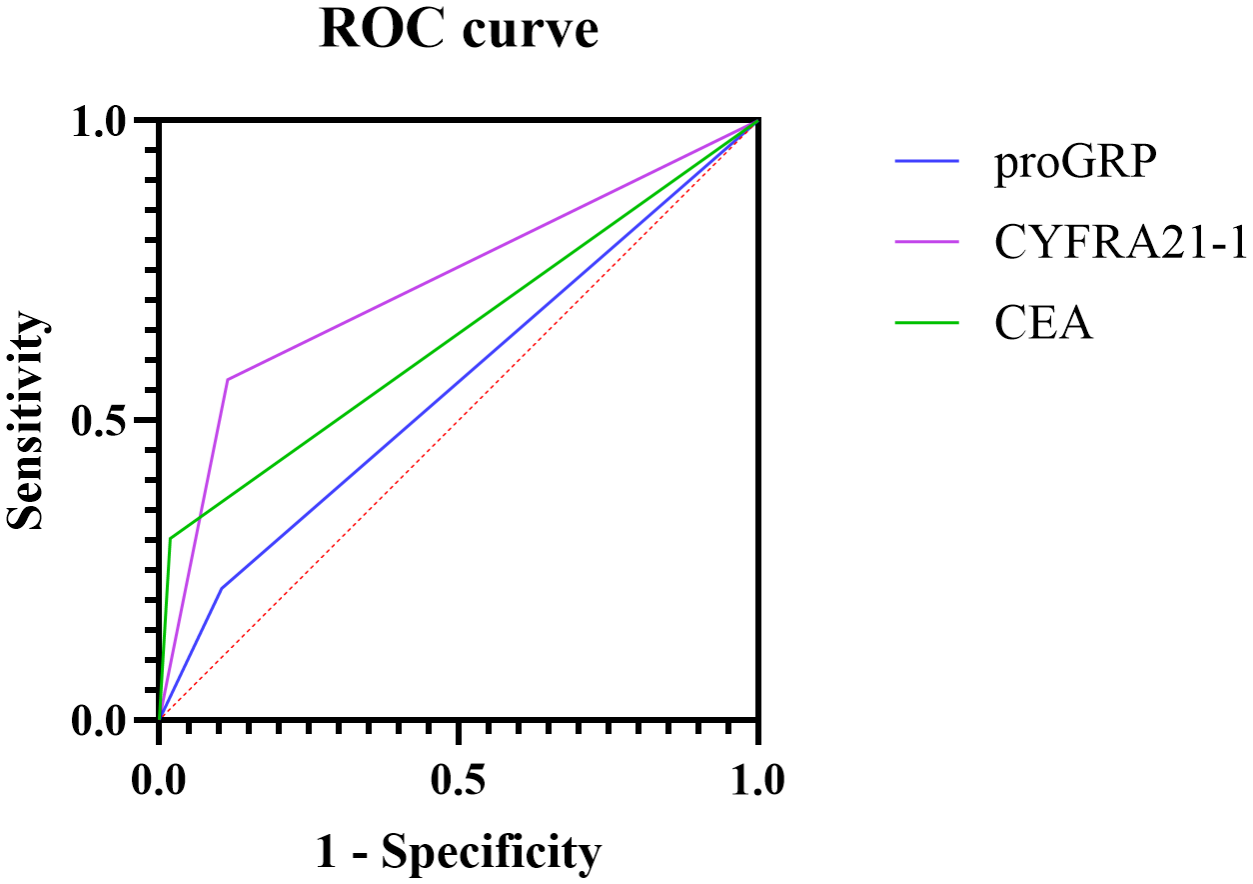
ROC curves of CEA, proGRP, and CYFRA21–1 in the auxiliary diagnosis of pleural effusion.

After plotting the ROC curve and calculating the AUC value (**Table 3**, **Figure 2**), it was found that CYFRA21–1 had the highest diagnostic efficacy for malignant pleural effusion among immunological tumor markers (AUC = 0.7269, 95% CI: 0.6734-0.7805), followed by CEA (AUC = 0.6420, 95% CI: 0.5852-0.6987). ProGRP had the lowest diagnostic efficacy (AUC = 0.5575, 95% CI: 0.4948-0.6202). NSE and SCC were found to have no discriminatory ability after calculation and were thus not included in **Table 3**.

**Table 3:** Comprehensive performance of CEA, proGRP, and CYFRA21–1 in the auxiliary diagnosis of pleural effusion.

AUC						
Tumor Markers	Value	95% CI	Sensitivity	Specificity	PPV	NPV
CEA	0.6420	0.5852-0.6987	30.3%	98.1%	97.6%	35.9%
proGRP	0.5575	0.4948-0.6202	22.0%	89.5%	84.1%	31.3%
CYFRA21-1	0.7269	0.6734-0.7805	56.8%	88.6%	92.6%	44.9%

AUC, area under the receiver operating characteristic curve; 95% CI, 95% confidence interval; PPV, positive predictive value (PPV = true positives/(true positives + false positives) × 100%); NPV, negative predictive value (NPV = true negatives/(true negatives + false negatives) × 100%).

In summary, CYFRA21–1 and CEA demonstrated the best overall performance for diagnosing malignant pleural effusions and were therefore included in the MTPC model.

### The diagnostic sensitivity of other indicators except tumor markers for malignant pleural effusion

This study further calculated and analyzed the sensitivities of PTGER4 gene methylation, SHOX2 gene methylation, combined PTGER4 and SHOX2 detection, DNA ploidy analysis, and cytological examination in the malignant pleural effusion group and the benign pleural effusion group. The sensitivities of PTGER4, SHOX2, PTGER4+SHOX2, DNA ploidy analysis, and cytology in the diagnosis of malignant pleural effusion were 40.5%, 50.4%, 65.9%, 23.5%, and 51.1%, respectively. The specificity of the combined PTGER4 and SHOX2 detection in benign pleural effusion was 92.4%. For malignant pleural effusions caused by different pathological types of tumors, the combined detection of the two genes had the highest diagnostic sensitivities in malignant pleural effusions caused by lung squamous cell carcinoma and small cell lung cancer, which were 90.5% and 90.0%, respectively. The highest diagnostic sensitivities of DNA ploidy analysis in malignant pleural effusions caused by lung squamous cell carcinoma and small cell lung cancer were 33.3% and 30.0%, respectively, with a specificity of 100%. The diagnostic sensitivities of cytomorphology in malignant pleural effusions caused by pleural mesothelioma and small cell lung cancer were the highest, which were 71.4% and 70.0%, respectively, with a specificity of 100% (**Table 4**).

**Table 4:** Comparison of the sensitivities of gene methylation, DNA ploidy, and cytology in the diagnosis of pleural effusion between patients with malignant pleural effusion and the benign pleural effusion group.

Histology subtype			PTGER4		SHOX2		PTGER4+SHOX2		DNA ploidy		Cytology	
		Total	n	Sensitivity	n	Sensitivity	n	Sensitivity	n	Sensitivity	n	Sensitivity
Malignant pleural effusion												
LUAC		187	68	36.4%	77	41.2%	109	58.3%	43	23.0%	97	51.9%
LUSC		21	10	47.6%	17	81.0%	19	90.5%	7	33.3%	9	42.9%
SCLC		10	9	90.0%	9	90.0%	9	90.0%	3	30.0%	7	70.0%
PM		14	4	28.6%	8	57.1%	10	71.4%	4	28.6%	10	71.4%
SC		1	1	100.0%	1	100.0%	1	100.0%	0	0.0%	0	0.0%
Unclassified Lung Cancer		31	15	48.4%	22	71.0%	26	83.9%	5	16.1%	12	38.7%
Total		264	107	40.5%	133	50.4%	174	65.9%	62	23.5%	135	51.1%
Benign pleural effusion												
PTB		67	2	3.0%	4	6.0%	6	9.0%	0	0.0%	0	0.0%
Non-tuberculous infection		38	0	0.0%	2	5.3%	2	5.3%	0	0.0%	0	0.0%
Total		105	2	1.9%	6	5.7%	8	7.6%	0	0.0%	0	0.0%

LUAC, lung adenocarcinoma; LUSC, lung squamous cell carcinoma; SCLC, small cell lung cancer; PM, pleural mesothelioma; SC, sarcomatoid carcinoma; PTB, pulmonary tuberculosis.

The number of patients with sarcomatoid carcinoma (SC) is small, and the sensitivity data should be interpreted with caution.

The sample sizes of the LUSC, SCLC, PM, and SC subgroups are relatively small, so their analytical results may be biased and should be interpreted with caution.

It should be noted that due to the small number of enrolled patients with SCLC and PM, the evaluation of sensitivity may be biased, and readers should interpret the results with caution.

### Evaluation of the performance of a MTPC panel for pleural effusion diagnosis

**Figure 3:**
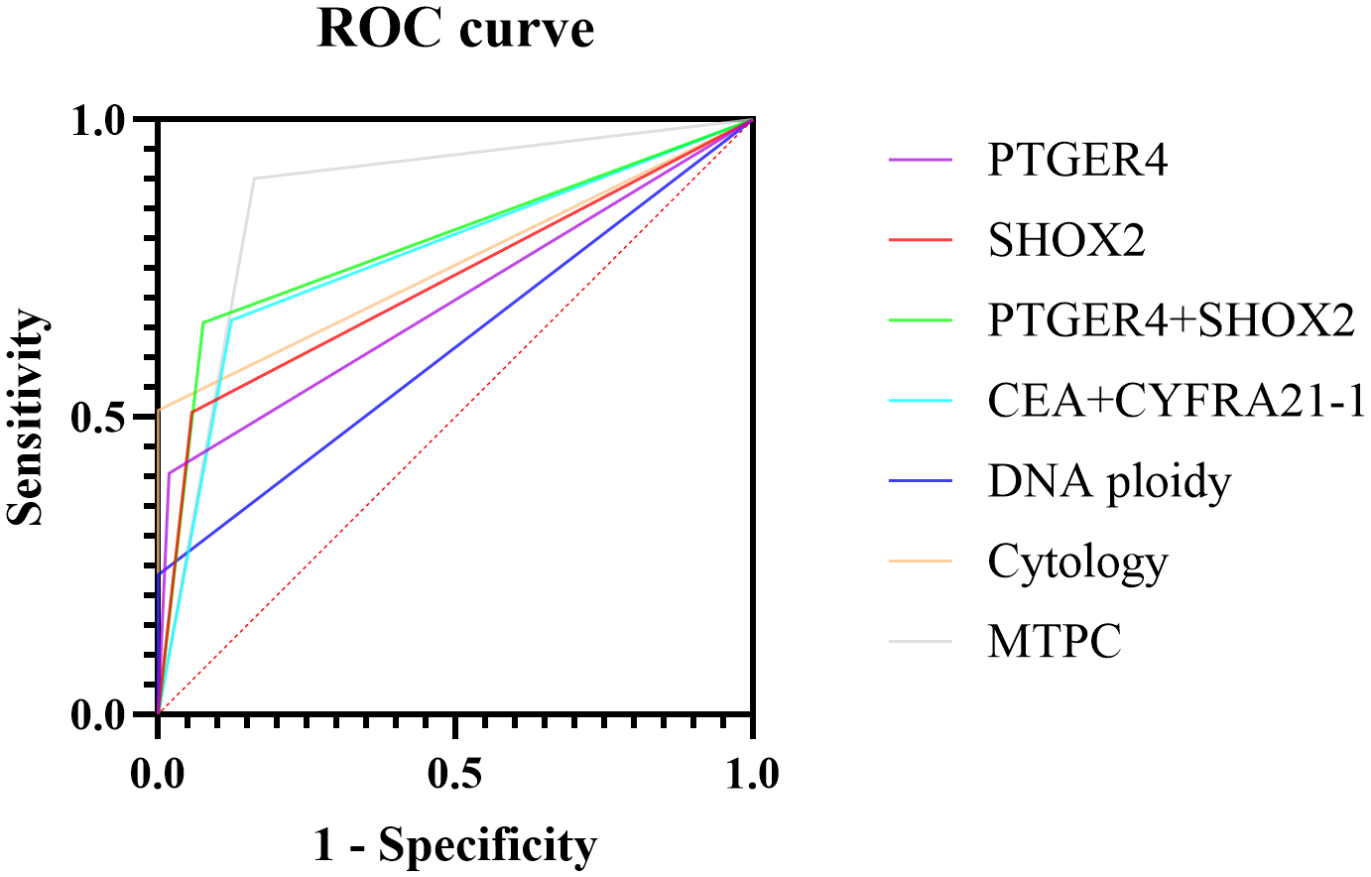
ROC curves of MTPC panel in the auxiliary diagnosis of pleural effusion. PTGER4 (95% CI: 0.6400 to 0.7462); SHOX2 (0.6734 to 0.7770); PTGER4+SHOX2 (0.7440 to 0.8389); CEA+CYFRA21-1 (0.7185 to 0.8206); DNA ploidy (0.5593 to 0.6755); Cytology (0.7084 to 0.8030); MTPC (0.8239 to 0.9157).

This study established a MTPC panel that integrated epigenetic molecular detection, immunological tumor markers, chromosomes, and cytomorphology, including PTGER4 + SHOX2 gene methylation, CEA + CYFRA21–1 tumor markers, DNA ploidy analysis, and cytological examination. The receiver operating characteristic curve (ROC curve) of all the above items was plotted, and the area under the curve (AUC) value was calculated (**Table 5**, **Figure 3**). The results showed that the diagnostic efficacy of the combined PTGER4 and SHOX2 detection was higher than that of single-gene methylation detection. The AUC of the combined detection was 0.7915 (95% CI: 0.7440-0.8389), with a sensitivity of 65.9% and a specificity of 92.4%. The AUC of the combined CEA and CYFRA21–1 detection was 0.7695 (95% CI: 0.7185-0.8206), with a sensitivity of 66.3% and a specificity of 87.6%, the diagnostic efficacy of the combined CEA and CYFRA21–1 detection was higher than that of single tumor marker detection. The AUCs of DNA ploidy analysis and cytomorphology were 0.6174 and 0.7557, respectively.

**Table 5:** Comprehensive performance of MTPC detection in the auxiliary diagnosis of pleural effusion.

	AUC					
	Value	95% CI	Sensitivity	Specificity	PPV	NPV
PTGER4	0.6931	0.6400-0.7462	40.5%	98.1%	98.2%	39.6%
SHOX2	0.7252	0.6734-0.7770	50.8%	94.3%	95.7%	43.2%
PTGER4+SHOX2	0.7915	0.7440-0.8389	65.9%	92.4%	95.6%	51.9%
CEA+CYFRA21-1	0.7695	0.7185-0.8206	66.3%	87.6%	93.1%	50.8%
DNA ploidy	0.6174	0.5593-0.6755	23.5%	100.0%	100.0%	34.2%
Cytology	0.7557	0.7084-0.8030	51.1%	100.0%	100.0%	44.9%
MTPC	0.8698	0.8239-0.9157	90.2%	83.8%	93.3%	77.2%

MTPC, M for methylation, T for tumor markers, P for ploidy, C for cytology.

This study innovatively proposed a diagnosis panel MTPC, aiming to provide clinicians with more comprehensive and multi-dimensional diagnostic information and thus reduce missed diagnoses. The results showed that MTPC had the best AUC performance, which was 0.8698 (95% CI: 0.8239-0.9157), with a sensitivity of 90.2% and a specificity of 83.8%. The AUC for MTPC was significantly higher than any single method (P<0.05). This indicated that the MTPC panel had a high diagnostic efficiency in the diagnosis of pleural effusion. Compared with single indicators or some combined indicators, it could more effectively distinguish the nature of pleural effusion.

Combined methylation detection and the CEA+CYFRA panel both outperformed single markers, while MTPC showed the best overall diagnostic accuracy.

### The supplementary diagnostic effect of the combined detection of methylation testing, tumor markers, and DNA ploidy on pleural effusions with an unclear cytological diagnosis

**Figure 4:**
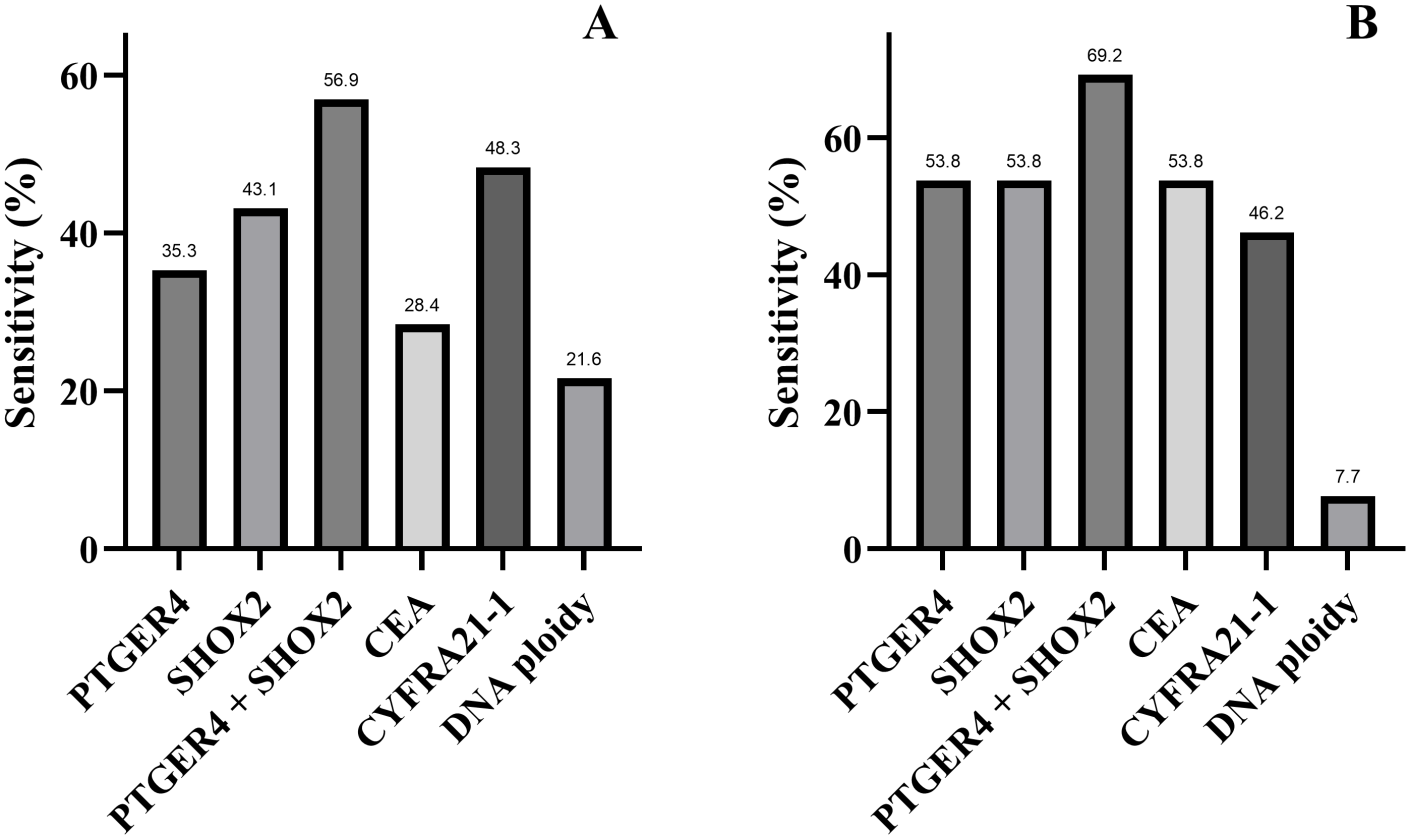
Sensitivities of gene methylation, tumor marker, and DNA ploidy detection in malignant pleural effusion with cytology undetermined **(A)** or negative **(B)** diagnosis.

**Figure 5:**
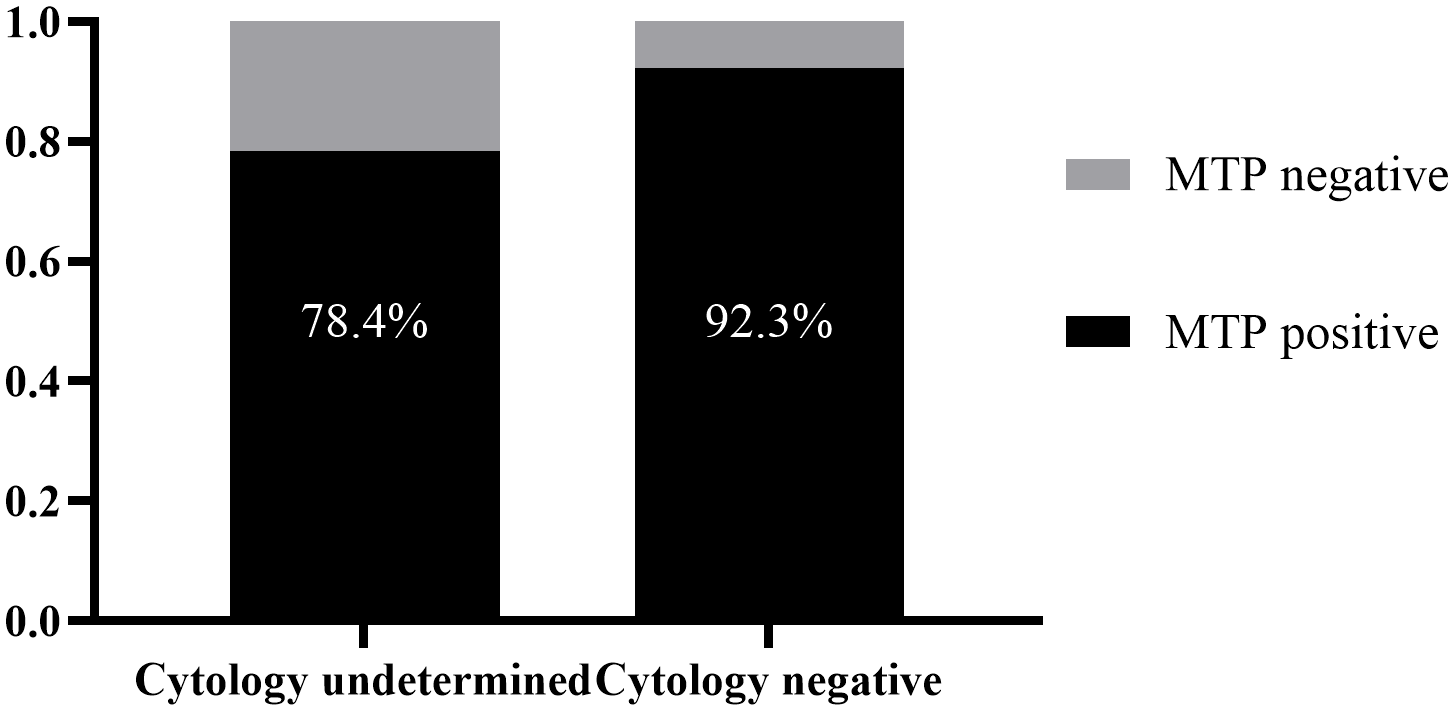
Combined detection sensitivity of gene methylation, tumor markers, and DNA ploidy in malignant pleural effusion patients stratified by cytology undetermined and cytology negative categories.

For patients with malignant pleural effusion whose cytopathological diagnosis was unclear, this study further analyzed the sensitivities of other diagnostic methods to evaluate the complementary role of the MTP panel in cytopathological diagnosis. Among patients with malignant pleural effusion whose cytopathological diagnosis was “cytology undetermined” (**Figure 4A**), the combined PTGER4 and SHOX2 detection had the highest sensitivity, reaching 56.9%. Followed by CYFRA21-1, with a sensitivity of 48.3%. The sensitivity of DNA ploidy analysis was 21.6%. For patients with malignant pleural effusion whose cytopathological diagnosis was “negative” (**Figure 4B**), the sensitivity of the combined PTGER4 and SHOX2 detection was 69.2%, the sensitivity of CEA was 53.8%, and the sensitivity of DNA ploidy analysis was 7.7%. When the MTP panel was applied, it could reduce the proportion of “cytology undetermined” reports by 78.4% and the proportion of missed diagnoses with “negative” reports by 92.3% (**Figure 5**). This indicated that the MTP panel could effectively compensate for the insufficient sensitivity of cytopathological diagnosis in determining the nature of pleural effusion. Especially when the cytopathological diagnosis gives a “gray-area” report such as “cytology undetermined”, the application of MTP panel can effectively reduce missed diagnoses.

## Discussion

When faced with pleural effusion highly suspected to be caused by malignant tumors, simply relying on traditional cytopathological diagnosis often yields unsatisfactory results. There are often benign reports of “negative” or ambiguous “gray-area” diagnoses such as “cytology undetermined”, which makes it impossible for clinicians to make a rapid diagnosis, thus delaying the treatment opportunity. Conversely, if the clinical suspicion is infection, but the pathological report indicates “cytology undetermined”, this not only shakes the clinician’s confidence in their own judgment but also, due to the inability to make a definite diagnosis based on the pathological results, forces repeated collection of pleural effusion samples for testing. During this period, patients can only receive routine treatment, increasing the risk of delayed disease progression. As a developing country, in addition to relatively high-level medical care in some cities, there are still varying degrees of gaps in primary medical care in most cities in China, especially in the field of pathological diagnosis, which requires a great deal of experience. Therefore, there is an urgent need for an objective, accurate, and rapid detection method to complement cytopathological diagnosis and provide more-dimensional evidence for the auxiliary diagnosis of pleural effusion. It is understood that gene methylation detection and DNA ploidy analysis are mainly carried out in large hospitals in mainland China, and public medical insurance policies vary across different provinces. Therefore, the data of this study focus on the performance of different diagnostic methods, and the MTPC combined diagnosis model needs to be referenced and adapted by the readers’ medical institutions according to their own conditions.

In this study, the proportion of males in the benign disease group was significantly higher, which may be due to the large number of enrolled tuberculosis patients, and tuberculosis has a higher incidence in males in China. Gender differences may to some extent affect the distribution of detection results, especially the statistics of immunological tumor markers. Additionally, a stratified analysis was conducted to compare the diagnostic performance of MTPC between patients of different genders. Statistical results showed that for sensitivity, the chi-square test of MTPC detection results across genders yielded χ²=2.37 (P = 0.123); for specificity, the chi-square test of MTPC detection results across genders gave χ²=1.09 (P = 0.344). Regarding the area under the receiver operating characteristic curve (AUC) - a core indicator of diagnostic efficacy - DeLong’s test was used to analyze AUC differences of MTPC between genders, resulting in P = 0.834. All the aforementioned statistical indicators had P-values greater than 0.05, indicating no significant difference in the diagnostic performance of MTPC among patients of different genders. However, as a single-center study, such an unbalanced gender distribution is unavoidable, and organizing multi-center studies will be the focus of our next work.

We first evaluated the diagnostic performance of immunological tumor markers in pleural effusion. After balancing sensitivity and specificity, we decided to detect CEA and CYFRA21–1 in pleural effusion. Furthermore, we excluded NSE, SCC, and proGRP from application in the diagnosis of pleural effusion. This selection was based on the data enrolled in the present study, which may carry a certain degree of data bias. Given that different research centers use immunological tumor marker detection reagents of varying brands, discrepancies may exist in the detection results. Thus, the selection of markers should be made in conjunction with the actual conditions of the research institution.

CEA is one of the most widely used tumor markers in clinical practice. However, in practical applications, it is found that the diagnostic specificity of CEA in blood is not high, as infections and inflammations often cause a transient increase in CEA levels in patients (9). In this study, although the sensitivity of CEA in pleural effusion was not high (30.3%), its specificity could reach 98.1%. One possible reason is that the sources of CEA in blood are relatively complex, and many tissues and organs may release CEA during systemic inflammatory reactions. In contrast, the tissue source of pleural effusion is relatively single, and the location of the lesion is limited. CEA released by tumor cells partly enters the peripheral blood circulation, while CEA in pleural effusion is released through the secretion and apoptosis of tumor cells at the lesion site, resulting in higher specificity.

Tumor gene methylation is an epigenetic tumor marker independent of cytomorphology and immunological detection, thus showing great potential to overcome the limitations of cytological examination. By detecting the genomic DNA of tumor-derived exfoliated cells, it is possible to determine whether specific tumor suppressor genes in patients are inactivated due to methylation, effectively improving the sensitivity of pathological diagnosis. In this study, when performing gene methylation detection on pleural effusion, we found that 9.0% of tuberculosis patients’ pleural effusion samples showed positive methylation signals for PTGER4 and SHOX2 genes. In another ongoing study on tuberculosis complicated with lung cancer, we also found that during bronchoscopy in tuberculosis patients, approximately 8.0% of the alveolar lavage fluid samples from tuberculosis patients showed positive methylation of SHOX2 and/or RASSF1A genes, and 6.9% of patients initially diagnosed with tuberculosis were also diagnosed with lung cancer simultaneously. Some studies have found that the probability of secondary lung cancer in patients with tuberculosis, especially active tuberculosis and refractory tuberculosis, is significantly increased (10, 11). Whether tuberculosis and lung cancer share similar epigenetic mechanisms needs further verification. However, these data suggest that in clinical practice, tuberculosis patients should not be diagnosed with a single-disease mindset. Tuberculosis and lung cancer share many similar clinical symptoms. When a diagnosis of tuberculosis is made, it is necessary to actively screen for the possibility of lung malignancies to avoid delaying the treatment opportunity.

After analyzing the correlation between methylation detection results and immunological tumor markers, we found an interesting phenomenon. The positive rate of the CYFRA21–1 tumor marker was significantly higher in patients with positive pleural effusion methylation than in those with negative methylation (p < 0.05). CYFRA21–1 is one of the important protein molecules that make up the cytoskeleton and is widely present in the cytoplasm of various epithelial cells, such as alveolar epithelial cells and bronchial epithelial cells (12, 13). SHOX2 is involved in the regulation of cell proliferation during tumorigenesis, including interacting with bone morphogenetic protein 4 (Bmp4) and activating the NF-κB signaling pathway (3). Abnormal cell proliferation leads to an accelerated renewal rate of the cytoskeleton, resulting in more CYFRA21–1 being released into the extracellular space.

This study also has limitations. For example, it is a retrospective single-center study, and all enrolled patients were inpatients. Patients with mild symptoms or those who opted for treatment at other hospitals were not included; thus, the representativeness of the study samples may not cover the entire population. For other institutions intending to adopt this diagnostic model, prospective studies or additional external validations are required to further confirm its applicability. Moreover, the enrolled benign disease samples are basically infectious diseases, which is because the clinical departments where the samples were sourced are mainly the Department of Respiratory Medicine and Department of Thoracic Surgery, not involving clinical departments such as the Department of Rheumatology and Department of Cardiology. This may lead to an imbalance in the distribution of benign samples. Therefore, the research conclusions may be more applicable to the differential diagnosis of lung cancer and infectious diseases. In addition, other biomarkers can be incorporated to compensate for missed diagnoses in patients by MTPC, and statistical methods such as logistic regression or machine learning can be combined to optimize the model, thereby improving the diagnostic accuracy. In future studies, we will consider combining methylation detection with efficacy evaluation, such as investigating the correlation between methylation detection and the therapeutic effects of demethylating drugs like azacitidine or decitabine.

As shown in the data presented in **Table 5**, the sensitivities of the immunological tumor markers, gene methylation, DNA ploidy analysis, and cytological examination included in this study all did not exceed 70%. However, after integrating them into the MTPC panel, the sensitivity increased to 90.2% while the specificity remained at 83.8%. This further indicates that the MTPC panel can compensate for the deficiencies in diagnostic efficacy of different individual diagnostic methods, reduce the disparities in diagnostic capabilities among medical institutions of varying levels, and thus deserves further promotion.

In this study, gene methylation, immunological tumor markers, chromosomal analysis, and cytopathological examination were combined for the auxiliary diagnosis of pleural effusion. Data in **Table 5** showed that the MTPC had an AUC value of 0.8698, a sensitivity of 90.2%, and a specificity of 83.8%. Results in **Figure 5** indicated that the MTPC panel effectively reduced the proportion of “gray-area” reports of “cytology undetermined” by 78.4% and the proportion of missed cytological examinations by 92.3%, providing more-dimensional evidence for clinical diagnosis.

## Data Availability

The original contributions presented in the study are included in the article/supplementary material. Further inquiries can be directed to the corresponding author.
